# Concurrent Occurrence of Primary Biliary Cirrhosis and Rheumatoid Arthritis

**DOI:** 10.7759/cureus.1562

**Published:** 2017-08-13

**Authors:** Stella Pak, Umar Darr, Zubair Khan, Andrew Kobalka, Zayd Safadi, Christine Dee

**Affiliations:** 1 Internal Medicine, University of Toledo Medical Center; 2 Department of Pathology, University of Toledo Medical Center; 3 College of Medicine and Life Sciences, University of Toledo Medical Center; 4 Wright State University Boonshoft School of Medicine

**Keywords:** rheumatoid arthritis, primary biliary cirrhosis

## Abstract

Primary biliary cirrhosis (PBC) is an autoimmune cholestatic disorder of the liver. A diagnostic serum marker for PBC is an anti-mitochondrial antibody. Most prominent histologic findings of PBC are portal inflammation and destruction of interlobular bile ducts. The PBC occurs only in 40 to 400 individuals per million in the general population. About 1.8 - 5.6% of individuals with this rare disorder have rheumatoid arthritis (RA). This case report describes a 56-year-old female with concurrent rheumatoid arthritis and primary biliary cirrhosis. The patients with RA are at higher risk of developing PBC compared to the general population. Thus, abnormal liver function test in the patients with RA, especially in the absence of alternative cause, warrants thorough investigation for PBC. Early diagnosis and treatment will improve the outcome of patients who develop PBC.

## Introduction

Rheumatoid arthritis (RA) is an autoimmune inflammatory disorder characterized by synovitis. The prevalence of RA is estimated to be about 0.5 - 1 % of the general population. In approximately two-third of individuals with RA, various types of liver histological abnormalities are found, including periportal fibrosis, portal tract inflammation, and sinusoidal dilatation. The lack of piecemeal and bridging necrosis or plasma cell infiltration are hallmarks of RA-associated liver disease [1]. The abnormalities, however, tend to be mild and have no clinical manifestation. 

Primary biliary cirrhosis (PBC) is an autoimmune cholestatic disorder of the liver. A diagnostic serum marker for PBC is an anti-mitochondrial antibody. Most prominent histologic findings of PBC are portal inflammation and destruction of interlobular bile ducts. The PBC occurs only in 40 to 400 individuals per million in the general population [2]. About 1.8 - 5.6% of individuals with this rare disorder have RA [3]. This concurrent manifestation of two rare diseases implies their pathophysiological association. Informed consent was obtained from the patient for this study.

## Case presentation

**Figure 1:**
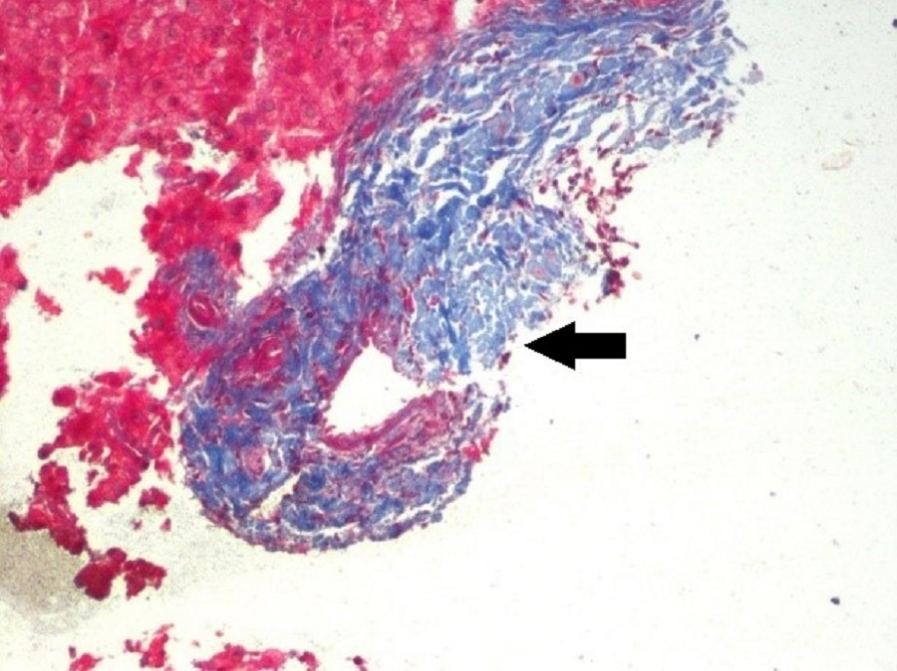
Liver biopsy showing increased collagen (black arrow) in portal areas with fibrous widening

**Figure 2:**
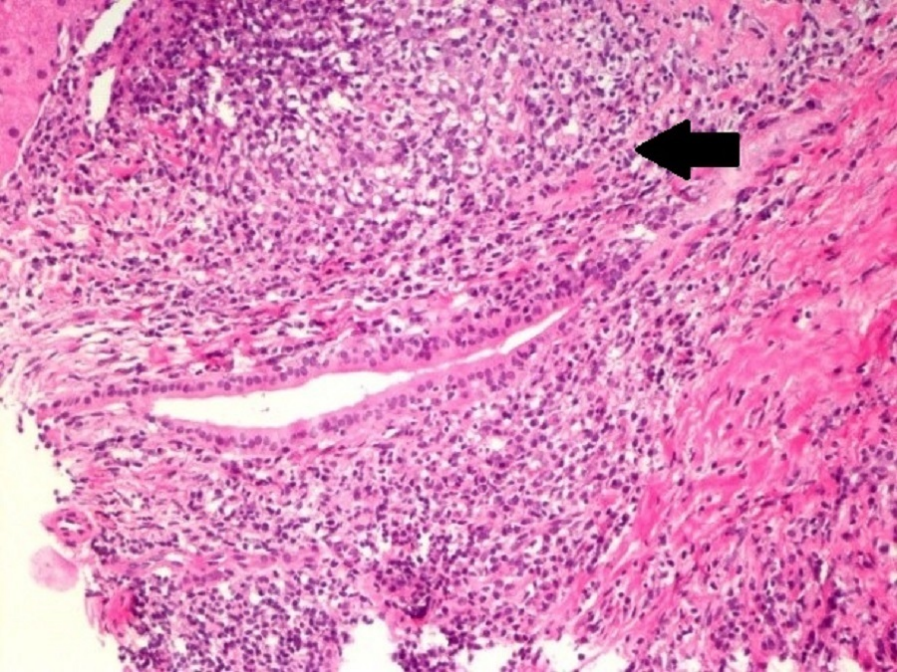
Mixed inflammatory infiltrate (black arrow) in portal areas with increased collagen and piecemeal necrosis

**Figure 3:**
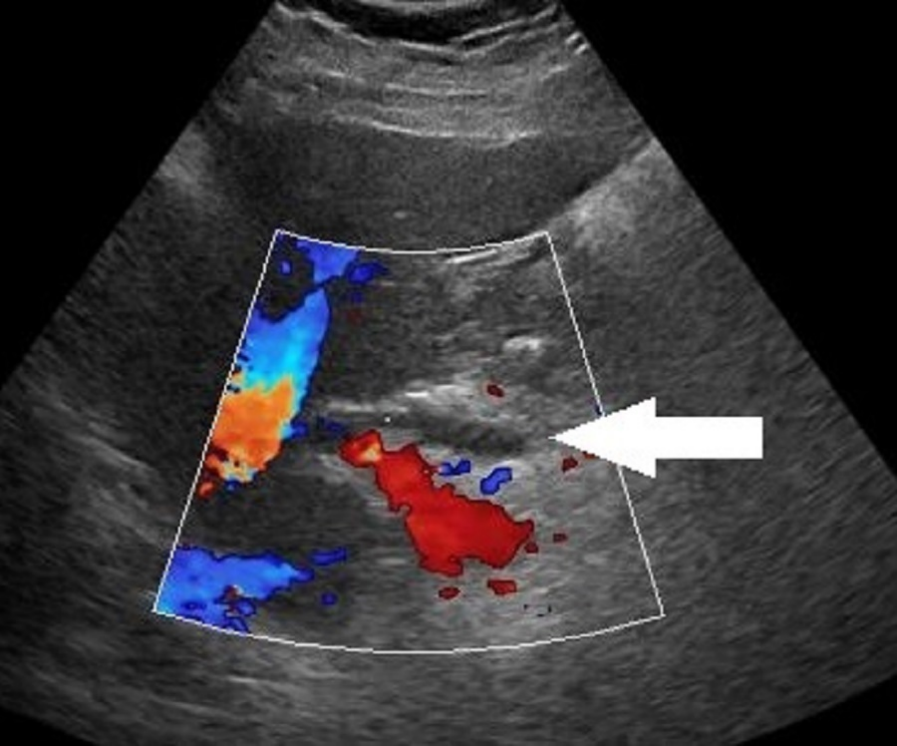
Hepatic ultrasound visualized mild heterogeneous increase (white arrow) in echogenicity of the liver, consistent with mild fibrotic change

A 56-year-old female with an 11-year history of RA was found to have mild elevations of aspartate aminotransferase (AST) of 54 U/L [normal values = 25 U/L] and alanine aminotransferase (ALT) of 49 U/L [normal values = 27 U/L] without evidence of hepatomegaly or ascites. Serologic testing revealed a 1:640 anti-mitochondrial antibody titer consistent with PBC. Hepatitis panel was negative. Liver biopsy showed increased collagen in portal areas with portal widening (Figure 1), mild piecemeal necrosis and a mixed inflammatory infiltrate (Figure 2). Hepatic ultrasound visualized mild heterogeneous increase in echogenicity of the liver, consistent with mild fibrotic change (Figure 3).

Ursodeoxycholic acid 250 mg twice daily was prescribed to treat the patients' PBC. While clinically asymptomatic over six months, the patients' liver function tests remained elevated (AST 36 U/L, ALT 43 U/L) due to prompted uptitration of ursodeoxycholic acid to 500 mg twice daily and as a result reducing her AST and ALT to normal limits.

## Discussion

The concurrent occurrence of RA and PBC in our patient supports the possible association between these two rare clinical entities. A postulated hypothesis for this association is genetic locus homogeneity, with the following genes found to be implicated in both disorders: histocompatibility complex, class II, DQ beta 1 ( HLA-DQB1), cytotoxic T-lymphocyte-associated protein 4 (CTLA4), membrane metallo-endopeptidase-like 1 (MMEL1), interferon regulatory factor 5 (IRF5), signal transducer and activator of transcription 4 (STAT4) and CXC chemokine receptor 5 ( CXCR5) [4].

About 50 – 60 % of individuals with PBC are asymptomatic at the time of diagnosis. However, if left untreated, they go on to develop symptoms such as pruritus, fatigue, and ascites [5]. It is reported that most patients with concurrent RA and PBC tend to develop RA years before PBC [6]. Being aware of the potential link between RA and PBC, it may be clinically warranted to evaluate RA patients with abnormal liver function test for PBC, even if they are asymptomatic.

## Conclusions

The patients with RA are at higher risk of developing PBC compared to the general population. Thus, abnormal liver function test in the patients with RA, especially in the absence of alternative cause, warrants thorough investigation for PBC. Early diagnosis and treatment will improve the outcome of patients who develop PBC.
